# Hypokalemic Quadriparesis As Initial Presentation of Secondary Sjogren Syndrome With Associated Autoimmune Thyroiditis: A Case Report

**DOI:** 10.7759/cureus.25420

**Published:** 2022-05-27

**Authors:** Faizan Fazal, Mohammad Ebad Ur Rehman, Saad Tahir, Ali Ahmad Ijaz, Saima Ambreen

**Affiliations:** 1 Internal Medicine, Rawalpindi Medical University, Rawalpindi, PAK; 2 Internal Medicine, Holy Family Hospital, Rawalpindi, PAK; 3 Medicine, Rawalpindi Medical University, Rawalpindi, PAK

**Keywords:** quadriparesis, autoimmune thyroid disorders, hypokalemia, distal renal tubular acidosis, sjögren’s syndrome

## Abstract

Sjögren's syndrome is an autoimmune disorder typically presenting as dry mouth and eyes (sicca syndrome). However, the disease can involve any organ, complicating diagnosis. Renal involvement may manifest as distal renal tubular acidosis, leading to hypokalemia. We report a case of a 25-year-old woman presenting with progressive quadriparesis and vomiting. She had severe hypokalemic paralysis due to distal renal tubular acidosis. The patient was diagnosed with secondary Sjögren syndrome with autoimmune thyroiditis. She recovered completely with potassium supplementation.

## Introduction

Sjögren's syndrome (SS) is a long-term autoimmune disorder that typically manifests as dry eyes and mouth (sicca syndrome). A consistent feature of SS is the presence of lymphocytic infiltration in the salivary glands. According to a meta-analysis in 2014, the global prevalence of SS was 6.92 cases per 100,000 person-years [1]. SS can be associated with other preexisting autoimmune diseases (secondary SS) or it can occur independently (primary SS).

Extra glandular manifestation of SS can involve any organ, thus complicating diagnosis and prognosis. Renal involvement is reported in up to 10% of cases, usually as tubulointerstitial nephritis [2]. Distal renal tubular acidosis (dRTA) is the most common renal manifestation. Complete dRTA (urinary acidification defect with acidosis) is less commonly reported compared to incomplete dRTA (urinary acidification defect without acidosis) [3]. Most cases of both complete and incomplete dRTA are asymptomatic or mild. Cases of severe muscle paralysis due to hypokalemia have been reported, however, usually in patients with primary SS. We report the case of a young female presenting with severe hypokalemic quadriparesis associated with metabolic acidosis, who was later diagnosed to be a case of secondary SS associated with autoimmune thyroid disease (AITD).

## Case presentation

A 25-year-old female presented with a three-day history of weakness of all four limbs and vomiting. The weakness was progressive, leading to complete paralysis of the upper and lower extremities by the third day of onset. She reported a history of dry mouth and dry eyes, with foreign body sensation in both eyes, for the past two months. Altered sensorium, urinary and bladder involvement, or convulsions were not reported with muscle paralysis. The patient denied a history of trauma, fall, fever, headache, or intake of diuretics, alcohols, or laxatives.

On examination, vital signs were within normal range and higher mental functions were intact. The patient had good dental hygiene but the oral cavity was dry. Motor power was 3/5 in upper and lower extremities on both sides and deep tendon reflexes were diminished bilaterally. On thyroid examination, the thyroid was diffusely enlarged, firm, and tender. Cardiovascular, respiratory, and gastrointestinal examinations were unremarkable.

Evaluation of serum electrolytes demonstrated severe hypokalemia (2.4 mEq/L). Arterial blood gases showed a picture of non-anion gap metabolic acidosis with a pH of 7.18, pCO_2_ 21.8 mmHg, HCO_3_ 8.1 mmol/L, sodium 141 mmol/L, chloride 121.5 mmol/L, and anion gap of 11.4 mmol/L. Renal function tests demonstrated a urea level of 80 mg/dL and a creatinine level of 2 mg/dL. These findings were consistent with dRTA. Her serological profile showed that anti-nuclear antibody, anti-Ro/SSA, and anti-La/SSB were positive. Schirmer's test was performed which was positive in both eyes. Thyroid function tests showed a low TSH level of 0.2 mIU/L, a high free T4 level of 13.5 mcg/dL, and a normal free T3 level of 320 pg/dL. The patient refused to consent to a salivary gland biopsy. Based on the patient’s history, examination, lab investigations, and autoantibody profile, a diagnosis of SS causing dRTA with associated AITD was made.

The patient responded well to potassium and bicarbonate supplementation, showing marked improvement in serum potassium levels and muscle paralysis. Serum potassium rose to 4.2 meq/L. She was discharged after three days of therapy on steroids and methimazole, with the advice of follow-up.

## Discussion

SS is an immune-mediated disease affecting the salivary and lacrimal glands and manifesting classically as keratoconjunctivitis sicca (dry eyes) and xerostomia (dry mouth). Sicca syndrome and SS are used synonymously as Henrik Sjögren studied this disease extensively and used the phrase “sicca syndrome” to describe it during his lifetime. However, present-day knowledge differentiates between the two, sicca syndrome being ocular and oral dryness due to any cause while SS is specifically defined as ocular and oral dryness due to immune-mediated destruction of salivary and lacrimal glands [4].

SS may occur in isolation or (more commonly) in association with other autoimmune conditions. The former is termed primary SS while the latter is termed secondary SS. Although this classification has been criticized by some sources, it is still widely used [5].

The pathogenesis of SS is not yet fully understood. Autoimmune activation of T and B lymphocytes is the most widely accepted explanation. The self-antigen to which this reaction takes place, however, remains obscure. Antibodies that have been found in patients with SS include SS-A (Ro), SS-B (La), rheumatoid factor, and ANAs. These, however, are not specific to the disease.

SS is associated with a wide variety of systemic manifestations, some of which are direct manifestations of the disease whereas others represent coincidental autoimmune diseases [4]. Renal involvement is common in SS, usually in the form of tubulointerstitial nephritis. Most cases, however, are either mild or asymptomatic [6]. dRTA with severe hypokalemia leading to quadriparesis, although rare, has been reported in several case reports as an initial presentation of SS [7-11]. This patient manifested similarly. The patient responded well to potassium supplementation.

The peculiar feature of this case was the involvement of the thyroid gland along with renal manifestations. This phenomenon of the coexistence of two or more autoimmune diseases is called polyautoimmunity [12]. The coexistence of these two entities is common, having been reported in over 20% of SS patients, and suggests a common genetic or environmental factor predisposition with similar pathogenesis [13,14]. This particular patient presented with hyperthyroidism. Hypothyroidism is a much more common presentation with SS [14], making this case rarer. A long-term follow-up study suggested that patients with primary SS are more likely to develop thyroid dysfunction [15]. However, a study also suggested that some patients with AITD show sicca symptoms that do not fulfill the criterion for SS and these patients are at an increased risk for developing SS [16]. So, it is unclear whether the SS in this patient was primary and thyroid dysfunction happened due to it or it was secondary being associated with thyroid dysfunction. The treatment modalities in both cases are similar. Secondary SS, although more common, has been much less researched and is often excluded from clinical trials [17]. More research is therefore needed to establish a definite cause-effect relationship between SS and thyroid dysfunction.

## Conclusions

Our patient presented with hypokalemic quadriparesis due to dRTA. The dRTA was a manifestation of SS, which was likely to be secondary as the patient also had hyperthyroidism-associated AITD. Autoimmune disorders must be considered while evaluating causes of dRTA, particularly in young females. SS should be a differential in such patients, even in the absence of sicca syndrome.
